# A Mathematical Study of the Influence of Hypoxia and Acidity on the Evolutionary Dynamics of Cancer

**DOI:** 10.1007/s11538-021-00914-3

**Published:** 2021-06-15

**Authors:** Giada Fiandaca, Marcello Delitala, Tommaso Lorenzi

**Affiliations:** Department of Mathematical Sciences “G. L. Lagrange”, Politecnico di Torino, Corso Duca degli Abruzzi, 24, Torino, Italy

**Keywords:** Mathematical oncology, Intra-tumour heterogeneity, Eco-evolutionary dynamics, Vascularised tumours, Partial integro-differential equations

## Abstract

Hypoxia and acidity act as environmental stressors promoting selection for cancer cells with a more aggressive phenotype. As a result, a deeper theoretical understanding of the spatio-temporal processes that drive the adaptation of tumour cells to hypoxic and acidic microenvironments may open up new avenues of research in oncology and cancer treatment. We present a mathematical model to study the influence of hypoxia and acidity on the evolutionary dynamics of cancer cells in vascularised tumours. The model is formulated as a system of partial integro-differential equations that describe the phenotypic evolution of cancer cells in response to dynamic variations in the spatial distribution of three abiotic factors that are key players in tumour metabolism: oxygen, glucose and lactate. The results of numerical simulations of a calibrated version of the model based on real data recapitulate the eco-evolutionary spatial dynamics of tumour cells and their adaptation to hypoxic and acidic microenvironments. Moreover, such results demonstrate how nonlinear interactions between tumour cells and abiotic factors can lead to the formation of environmental gradients which select for cells with phenotypic characteristics that vary with distance from intra-tumour blood vessels, thus promoting the emergence of intra-tumour phenotypic heterogeneity. Finally, our theoretical findings reconcile the conclusions of earlier studies by showing that the order in which resistance to hypoxia and resistance to acidity arise in tumours depend on the ways in which oxygen and lactate act as environmental stressors in the evolutionary dynamics of cancer cells.

## Introduction

Cancer is a dynamic disease, the characteristics of which are constantly evolving. This is reflected in the fact that the genotypic and phenotypic properties of cancer cells may change across space and time within the same tumour, and the dynamics of tumours with the same histological features are still likely to vary across patients. Moreover, since the same cancer clones may arise through different evolutionary pathways, the fact that two tumours have a similar clonal composition at a given point in time does not necessarily indicate that they share similar evolutionary histories, and does not rule out the possibility that their future evolution will diverge significantly (Maley et al. 2017). These sources of variability within and between tumours provide the substrate for the emergence and development of intra- and inter-tumour heterogeneity, which are major obstacles to cancer eradication (Gillies et al. 2012; Marusyk et al. 2012).

Clinical evidence suggests that cancer cells and the tumour microenvironment mutually shape each other (Gallaher et al. 2019). This supports the idea that tumours can be seen as evolving ecosystems where cancer cells with different phenotypic characteristics proliferate, die, undergo genotypic and phenotypic changes, and compete for space and resources under the selective pressure exerted by the various components of the tumour microenvironment (Gallaher and Anderson 2016; Gay et al. 2016; Ibrahim-Hashim et al. 2017; Korolev et al. 2014; Lloyd et al. 2016; Loeb 2001; Merlo et al. 2006; Michor and Polyak 2010; Villa et al. 2021; Vander Linden and Corbet 2020). In this light, intra-tumour phenotypic heterogeneity can be regarded as the outcome of an eco-evolutionary process in which spatial variability of the concentration of abiotic factors (i.e. substrates and metabolites) across the tumour supports the formation of distinct ecological niches whereby cells with different phenotypic characteristics may be selected (Casciari et al. 1992; Gatenby et al. 2007; Hockel and Vaupel 2001).

In normal tissues, cells produce the energy required to sustain their proliferation via oxidative phosphorylation (i.e. they rely on oxygen as their primary source of energy) and turn to glycolysis only when oxygen is scarce. In tumours, the presence of hypoxic regions (i.e. regions where the oxygen levels are below the physiological ones) induces cells to transiently switch to a glycolytic metabolic phenotype (i.e. to rely on glucose as their primary source of energy) (Vaupel et al. 2001). Cancer cells eventually acquire such a glycolytic phenotype and express it also in aerobic conditions, leading to the so-called Warburg effect (Kim and Dang 2006). The interplay between the high glycolytic rate of cancer cells and low perfusion in tumours brings about accumulation of lactate (i.e. a waste product of glycolysis), which causes acidity levels in the tumour microenvironment to rise (i.e. the pH level drops) (Tang et al. 2012).

Since hypoxia and acidity act as environmental stressors promoting selection for cancer cells with a more aggressive phenotype (Robertson-Tessi et al. 2015; Tang et al. 2012), an in-depth theoretical understanding of the spatio-temporal processes that drive the adaptation of tumour cells to hypoxic and acidic microenvironments may open up new avenues of research in oncology and cancer treatment (Martinez-Outschoorn et al. 2016). In this regard, mathematical models can be an important source of support to cancer research, as they enable extrapolation beyond scenarios which can be investigated through experiments and may reveal emergent phenomena that would otherwise remain unobserved (Anderson and Quaranta 2008; Byrne 2010; Chaplain 2020; Chisholm et al. 2016; Eastman et al. 2020; Hamis and Powathil 2020; Poleszczuk et al. 2015). For instance, in their pioneering paper (Gatenby and Gawlinski 1996), Gatenby and Gawlinski used a reaction–diffusion system to explore how nonlinear interactions between cancer cells and abiotic components of the tumour microenvironment may shape tumour growth. The Gatenby–Gawlinski model has recently been extended in Strobl et al. (2020), in order to take into account the presence of cells with different phenotypic characteristics within the tumour. Hybrid cellular automaton models have been employed to study the impact of hypoxia and acidity on tumour growth and invasion (Anderson et al. 2006, 2009; Gatenby et al. 2007; Hatzikirou et al. 2012; Kim et al. 2018; Robertson-Tessi et al. 2015). A mechanical model of tumour growth whereby cells are allowed to switch between aerobic and anaerobic metabolism was presented in Astanin and Preziosi (2009). Integro-differential equations and partial integro-differential have been used in Ardaševa et al. (2020a), Chaplain et al. (2021), Lorenzi et al. (2018), Lorz et al. (2015), Villa et al. (2021) to investigate the ecological role of hypoxia in the development of intra-tumour phenotypic heterogeneity.

In this paper, we complement these earlier studies by presenting a mathematical model to study the influence of hypoxia and acidity on the evolutionary dynamics of cancer cells in vascularised tumours. The model comprises a system of partial integro-differential equations that describe the phenotypic evolution of cancer cells in response to dynamic variations in the spatial distribution of three abiotic factors that are key players in tumour metabolism: oxygen, glucose and lactate.

The remainder of the paper is organised as follows: In Sect. 2, we present the model equations and the underlying modelling assumptions. In Sect. 3, we summarise the main results of numerical simulations of the model and discuss their biological implications. Section 4 concludes the paper and provides a brief overview of possible research perspectives.

## Model Description

**Fig. 1:**
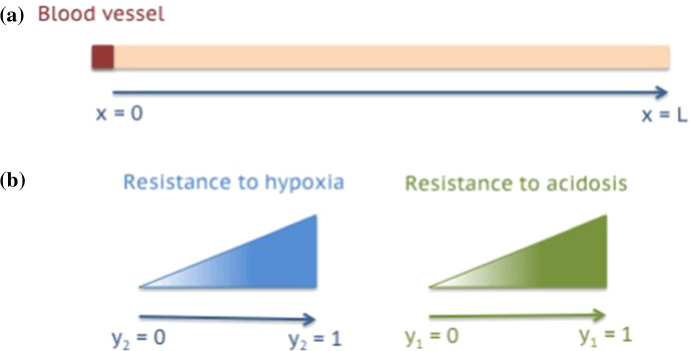
**a** Schematic of the spatial domain defined as a one-dimensional region of vascularised tissue of length $\mathrm{L}$. A blood vessel is assumed to be present at $x=0$. **b** Schematics illustrating the relationships between the values of the variables $y_2$ and $y_1$ modelling the phenotypic state of tumour cells and the levels of resistance to hypoxia and acidosis (Color figure online)

We consider a one-dimensional region of vascularised tissue of length $\mathrm{L} > 0$. We describe the spatial position of every tumour cell in the tissue region by a scalar variable $x \in [0,\mathrm{L}]$, and we assume a blood vessel to be present at $x=0$ (cf. the schematic in Fig. 1a). Moreover, building upon the modelling framework developed in Chaplain et al. (2021), Lorenzi et al. (2018), Lorz et al. (2015), Villa et al. (2021), we model the phenotypic state of every cell by a vector $\mathbf{y} = (y_1,y_2) \in [0,1]^2$ (cf. the schematics in Fig. 1b). Here, $y_1 \in [0,1]$ represents the normalised level of expression of an acidity-resistant gene (e.g. the LAMP2 gene), while $y_2 \in [0,1]$ represents the normalised level of expression of a hypoxia-resistant gene (e.g. the GLUT-1 gene) (Damaghi et al. 2015; Gatenby et al. 2007).

We describe the phenotypic distribution of tumour cells at position *x* and time $t \in [0,\mathrm{T}]$, with $\mathrm{T}>0$, by means of the local population density function $n(t,x,\mathbf{y})$ (i.e. the local phenotypic distribution of tumour cells). We define the cell density $\rho (t,x)$, the local mean level of expression of the acidity-resistant gene $\mu _1(t,x)$ and the local mean level of expression of the hypoxia-resistant gene $\mu _2(t,x)$ as

1 $$\begin{aligned} \rho (t,x):=\int _{[0,1]^2} n(t,x,\mathbf{y}) \,\mathrm{d}{} \mathbf{y}, \quad \mu _i(t,x):=\frac{1}{\rho (t,x)}\int _{[0,1]^2} y_i\,n(t,x,\mathbf{y})\,\mathrm{d}{} \mathbf{y} \end{aligned}$$

for $i=1,2$. Moreover, we define the phenotypic distribution of tumour cells across the whole tissue region $f(t,\mathbf{y})$ as the mean value of $n(t,x,\mathbf{y})$ on the interval $[0,\mathrm{L}]$, i.e.

2 $$\begin{aligned} f(t,\mathbf{y}) := \frac{1}{\mathrm{L}} \, \int _0^{\mathrm{L}} n(t,x,\mathbf{y}) \, \mathrm{d}x. \end{aligned}$$

Similarly, we define the levels of expression of the acidity-resistant gene and the hypoxia-resistant gene across the whole tissue region as the mean values of $\mu _1(t,x)$ and $\mu _2(t,x)$ on the interval $[0,\mathrm{L}]$, respectively, i.e.

3 $$\begin{aligned} \nu _1(t) := \frac{1}{\mathrm{L}} \, \int _0^{\mathrm{L}} \mu _1(t,x) \, \mathrm{d}x \quad \text {and} \quad \nu _2(t) := \frac{1}{\mathrm{L}} \, \int _0^{\mathrm{L}} \mu _2(t,x) \, \mathrm{d}x. \end{aligned}$$

The local concentrations of oxygen, glucose and lactate at position *x* and time *t* are denoted by $S_o(t,x)$, $S_g(t,x)$ and $S_l(t,x)$, respectively.

### Dynamics of Tumour Cells

We describe the dynamics of tumour cells through the following balance equation for the local population density function $n(t,x,\mathbf{y})$

4 $$\begin{aligned} \frac{\partial n}{\partial t} = \underbrace{\beta _n \frac{\partial ^2 n}{\partial x^2}}_{\begin{array}{c} \text {undirected, random}\\ \text {cell movement} \end{array}}+ \underbrace{\theta \, \varDelta _{\mathbf{y}} n}_{\begin{array}{c} \text {spontaneous}\\ \text {phenotypic changes} \end{array}} + \;\, \underbrace{ R\,(S_o,S_g,S_l,\rho ,\mathbf{y})\,n}_{\text {proliferation and death}}, \end{aligned}$$

with $(t,x,\mathbf{y}) \in (0,\mathrm{T}] \times (0,\mathrm{L}) \times (0,1)^2$, subject to suitable initial conditions. We complement (4) with zero-flux boundary conditions at $x=0$ and $x=\mathrm{L}$ (i.e. we assume that cells cannot leave the tissue region) and zero-flux boundary conditions on the boundary of the square $[0,1]^2$ (i.e. we assume that cells cannot have normalised levels of gene expression smaller than 0 or larger than 1).

The first term on the right-hand side of the partial integro-differential equation (4) models the effect of undirected, random movement, which is described through Fick’s first law of diffusion with diffusivity $\beta _n>0$.

The second term on the right-hand side of the partial integro-differential equation (4) models the effect of heritable, spontaneous phenotypic changes, which occur at rate $\theta >0$. Similar diffusion terms have been used in a number of previous papers to model the effect of spontaneous phenotypic changes (Alfaro and Veruete 2019; Almeida et al. 2019; Ardaševa et al. 2020b; Bouin et al. 2012; Chisholm et al. 2015; Iglesias and Mirrahimi 2018; Genieys et al. 2006; Lorenzi et al. 2016; Mirrahimi and Gandon 2020; Perthame and Génieys 2007) and can be obtained as the deterministic continuum limit of corresponding stochastic individual-based models in the asymptotic regime of large numbers of individuals and small phenotypic changes (Champagnat et al. 2006; Chisholm et al. 2016).

The function $R(S_o,S_g,S_l,\rho ,\mathbf{y})$ represents the fitness of tumour cells in the phenotypic state $\mathbf{y}$ at position *x* and time *t* under the environmental conditions given by the concentrations of abiotic factors $S_o(t,x)$, $S_g(t,x)$ and $S_l(t,x)$, and the cell density $\rho (t,x)$ (i.e. $R(S_o,S_g,S_l,\rho ,\mathbf{y})$ is the phenotypic fitness landscape of the tumour). We use the following definition

5 $$\begin{aligned} R(S_o,S_g,S_l,\rho ,\mathbf{y}) \; := \underbrace{P(S_o,S_g, y_2)}_{\begin{array}{c} \text {proliferation and}\\ \text {death due to}\\ \text {oxygen-driven selection} \end{array}} - \underbrace{D(S_l,y_1)}_{\begin{array}{c} \text {death due to}\\ \text {lactate-driven selection} \end{array}} - \underbrace{d(\rho ).}_{\begin{array}{c} \text {death due to}\\ \text {competition}\\ \text {for space} \end{array}} \end{aligned}$$

Here, the function $P(S_o,S_g, y_2)$ is the rate at which cells with level of expression $y_2$ of the hypoxia-resistant gene proliferate via oxidative phosphorylation and glycolysis and die due to oxygen-driven selection (i.e. $P(S_o,S_g, y_2)$ is a net proliferation rate). The function $D(S_l,y_1)$ is the rate at which cells with level of expression $y_1$ of the acidity-resistant gene die due to lactate-driven selection. The function $d(\rho )$ models the rate of cell death due to competition for space associated with saturation of the cell density.

#### Modelling Oxygen-Driven Selection

Based on the theoretical results and experimental data presented in Korolev et al. (2014), Vaupel et al. (2001), we focus on a scenario corresponding to the following biological assumptions.

##### Assumption 1

There exist two threshold levels of the oxygen concentration $O_M>O_m>0$ such that the environment surrounding the cells is: hypoxic if $S_o \le O_m$; moderately oxygenated if $O_m< S_o < O_M$; normoxic (i.e. well oxygenated) if $S_o \ge O_M$.

##### Assumption 2

Cells proliferate at a rate that depends on the concentrations of oxygen and glucose. Moreover, the trade-off between increase in cell death associated with sensitivity to hypoxia and decrease in cell proliferation associated with acquisition of resistance to hypoxia results in the existence of a level of expression of the hypoxia-resistant gene which is the fittest in that: a lower level of gene expression would correlate with a lower resistance to hypoxia and thus a higher death rate; a higher level of gene expression would correlate with a larger fitness cost and thus a lower proliferation rate. Cells with levels of gene expression that are closer to the fittest one are more likely to survive than the others. Hence, the farther the gene expression level of a cell is from the fittest one, the more likely is that the cell will die due to a form of oxygen-driven selection.

##### Assumption 3

In normoxic environments (i.e. when $S_o \ge O_M$), the energy required for cell proliferation is produced via oxidative phosphorylation and the cell proliferation rate is a monotonically increasing function of the concentration of oxygen. In hypoxic environments (i.e. when $S_o \le O_m$), the energy required for cell proliferation is produced via glycolysis and the cell proliferation rate is a monotonically increasing function of the concentration of glucose. In moderately oxygenated environments (i.e. when $O_m< S_o < O_M$), the energy required for cell proliferation is produced via both oxidative phosphorylation and glycolysis. Moreover, the cell proliferation rate is a monotonically increasing function of the concentrations of oxygen and glucose, and lower values of the oxygen concentration correlate with a greater tendency of the cells to proliferate via glycolysis.

##### Assumption 4

The fittest level of expression of the hypoxia-resistant gene (i.e. the gene associated with the variable $y_2$) may vary with the oxygen concentration. In particular: in normoxic environments (i.e. when $S_o \ge O_M$), the fittest level of gene expression is the minimal one (i.e. $y_2=0$); in hypoxic environments (i.e. when $S_o \le O_m$) the fittest level of gene expression is the maximal one (i.e. $y_2=1$); in moderately oxygenated environments (i.e. when $O_m< S_o < O_M$), the fittest level of gene expression is a monotonically decreasing function of the oxygen concentration (i.e. it decreases from $y_2=1$ to $y_2=0$ when the oxygen concentration increases).

Under Assumptions 1 and 2, we define the net proliferation rate $P(S_o,S_g, y_2)$ as

6 $$\begin{aligned} P(S_o,S_g, y_2) \; := \underbrace{p_o(S_o)}_{\begin{array}{c} \text {proliferation via}\\ \text {oxidative phosphorylation} \end{array}} + \underbrace{p_g(S_o,S_g)}_{\begin{array}{c} \text {proliferation via}\\ \text {glycolysis} \end{array}} - \underbrace{\eta _o \, \big (y_2-\varphi _o(S_o)\big )^2}_{\begin{array}{c} \text {death due to}\\ \text {oxygen-driven selection} \end{array}}. \end{aligned}$$

In (6), the function $p_o(S_o)$ models the rate of cell proliferation via oxidative phosphorylation, while the function $p_g(S_o,S_g)$ models the rate of cell proliferation via glycolysis. Furthermore, the third term in the definition given by (6) is the rate of death induced by oxygen-driven selection. Here, the parameter $\eta _o>0$ is a selection gradient that quantifies the intensity of oxygen-driven selection and the function $\varphi _o(S_o)$ is the fittest level of expression of the hypoxia-resistant gene under the environmental conditions given by the oxygen concentration $S_o$.

##### Remark 1

In (6), the distance between $y_2$ and $\varphi _o(S_o)$ is computed as $\big (y_2-\varphi _o(S_o)\big )^2$. Alternatively, one could compute such a distance as $\big |y_2-\varphi _o(S_o)\big |$. However, we have chosen $\big (y_2-\varphi _o(S_o)\big )^2$ over $\big |y_2-\varphi _o(S_o)\big |$ because, as discussed in Ardaševa et al. (2020a), Lorenzi et al. (2016), it leads to a smoother fitness function which is closer to the approximate fitness landscapes which can be inferred from experimental data through regression techniques.

Under Assumptions 3 and 4, we use the definitions of the functions $p_o(S_o)$, $p_g(S_o,S_g)$ and $\varphi _o(S_o)$ given hereafter

7 $$\begin{aligned} p_o(S_o) := \dfrac{\gamma _o\,S_o}{\alpha _o+S_o}\,w(S_o), \qquad p_g(S_o,S_g) := \dfrac{\gamma _g\,S_g}{\alpha _g+S_g}\,\big (1-w(S_o)\big ), \end{aligned}$$

with

8 $$\begin{aligned} w(S_o):= {\left\{ \begin{array}{ll} \begin{array}{ll} 1 &{}\qquad S_o \ge O_M\\ \\ \displaystyle 1 - \frac{O_M-S_o}{O_M-O_m} &{} \qquad O_m< S_o < O_M\\ \\ \displaystyle 0 &{} \qquad S_o \le O_m \end{array} \end{array}\right. } \end{aligned}$$

and

9 $$\begin{aligned} \varphi _o(S_o):= {\left\{ \begin{array}{ll} \begin{array}{ll} 0 &{} \qquad S_o \ge O_M\\ \\ \displaystyle \frac{O_M-S_o}{O_M-O_m} &{}\qquad O_m< S_o < O_M\\ \\ \displaystyle 1 &{} \qquad S_o \le O_m. \end{array} \end{array}\right. } \end{aligned}$$

**Fig. 2:**
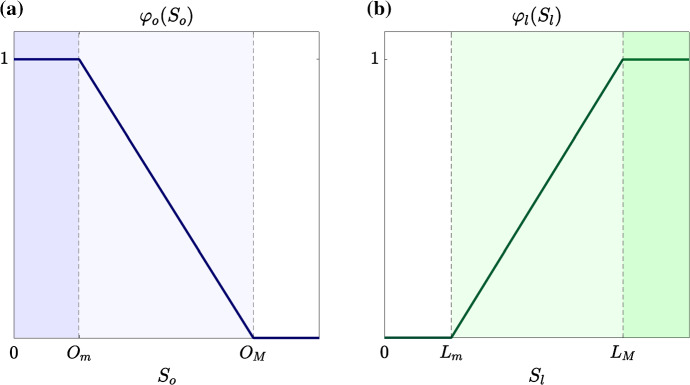
**a** Plot of the fittest level of expression of the hypoxia-resistant gene $\varphi _o(S_o)$ defined via (9). The vertical, dashed lines highlight the oxygen levels $O_m$ and $O_M$. Hence, the white region corresponds to normoxic conditions, the pale-blue region corresponds to moderately oxygenated environments and the blue region corresponds to hypoxic conditions. **b** Plot of the fittest level of expression of the acidity-resistant gene $\varphi _l(S_l)$ defined via (11). The vertical, dashed lines highlight the lactate levels $L_m$ and $L_M$. Hence, the white region corresponds to mildly acidic conditions, the pale-green region corresponds to moderately acidic conditions and the green region corresponds to highly acidic conditions (Color figure online)

In (7), the parameters $\gamma _o>0$ and $\gamma _g>0$ model the maximum rates of cell proliferation via oxidative phosphorylation and glycolysis, respectively. The parameters $\alpha _o>0$ and $\alpha _g>0$ are the Michaelis–Menten constants of oxygen and glucose. The weight function $w(S_o)$ defined via (8) ensures that Assumption 3 is satisfied, while definition (9) of $\varphi _o(S_o)$ is such that Assumption 4 is satisfied (cf. the plot in Fig. 2a).

#### Modelling Lactate-Driven Selection

Based on theoretical results and experimental data presented in Robertson-Tessi et al. (2015), we focus on a scenario corresponding to the following biological assumptions.

##### Assumption 5

There exist two threshold levels of the lactate concentration $L_M>L_m>0$ such that the environment surrounding the cells is: mildly acidic if $S_l \le L_m$; moderately acidic if $L_m< S_l < L_M$; highly acidic if $S_l \ge L_M$.

##### Assumption 6

Cells die at a rate that depends on the concentration of lactate. Moreover, the trade-off between increase in cell death associated with sensitivity to acidity and decrease in cell proliferation associated with acquisition of resistance to acidity results in the existence of a level of expression of the acidity-resistant gene which is the fittest in that: a lower level of gene expression would correlate with a lower resistance to acidity and thus a higher death rate; a higher level of gene expression would correlate with a larger fitness cost and thus a lower proliferation rate. Cells with levels of gene expression that are closer to the fittest one are more likely to survive than the others. Hence, the farther the gene expression level of a cell is from the fittest one, the more likely is that the cell will die due to a form of lactate-driven selection.

##### Assumption 7

The fittest level of expression of the acidity-resistant gene (i.e. the gene associated with the variable $y_1$) may vary with the lactate concentration. In particular: in mildly acidic environments (i.e. when $S_l \le L_m$), the fittest level of gene expression is the minimal one (i.e. $y_1=0$); in highly acidic environments (i.e. when $S_l \ge L_M$) the fittest level of gene expression is the maximal one (i.e. $y_1=1$); in moderately acidic environments (i.e. when $L_m< S_l < L_M$), the fittest level of gene expression is a monotonically increasing function of the lactate concentration (i.e. it increases from $y_1=0$ to $y_1=1$ when the lactate concentration increases).

Under Assumptions 5 and 6, we define the rate of cell death due to lactate-driven selection $D(S_l,y_1)$ as

10 $$\begin{aligned} D(S_l, y_1) := \eta _l \, \big (y_1-{\varphi _l(S_l)}\big )^2. \end{aligned}$$

In (10), the parameter $\eta _l>0$ is a selection gradient that quantifies the intensity of lactate-driven selection and the function $\varphi _l(S_l)$ is the fittest level of expression of the acidity-resistant gene under the environmental conditions given by the lactate concentration $S_l$. Considerations analogous to those made in Remark 1 on the term $\big (y_2-\varphi _o(S_o)\big )^2$ in (6) apply to the term $\big (y_1-\varphi _l(S_l)\big )^2$ in (10). Finally, we use the definition of the function $\varphi _l(S_l)$ given hereafter (cf. the plot in Fig. 2b), so that Assumption 7 is satisfied:

11 $$\begin{aligned} \varphi _l (S_l):={\left\{ \begin{array}{ll} \begin{array}{ll} \displaystyle 0 &{} \qquad S_l \le L_m\\ \\ \displaystyle \frac{S_l-L_m}{L_M-L_m}&{} \qquad L_m< S_l < L_M\\ \\ \displaystyle 1 &{} \qquad S_l \ge L_M. \end{array} \end{array}\right. } \end{aligned}$$

#### Modelling Competition for Space

We define the rate of cell death due to competition for space associated with saturation of the cell density as

12 $$\begin{aligned} d(\rho ) := \kappa \, \rho . \end{aligned}$$

Here, the proportionality constant $\kappa >0$ is related to the local carrying capacity of the tumour, which may vary depending on the tumour type.

### Dynamics of Abiotic Factors

Oxygen and glucose are consumed by tumour cells, while lactate is produced by tumour cells as a waste product of glycolysis. Moreover, oxygen, glucose and lactate diffuse in space and decay over time. Hence, their dynamics are governed by the following balance equations for the functions $S_o(t,x)$, $S_g(t,x)$ and $S_l(t,x)$, respectively,

13 $$\begin{aligned}&\frac{\partial S_o}{\partial t} \; = \; \underbrace{\beta _o\frac{\partial ^2 S_o}{\partial x^2}}_{\begin{array}{c} \text {diffusion} \end{array}} \quad - \; \underbrace{\lambda _o\,S_o}_{\begin{array}{c} \text {natural} \\ \text {decay} \end{array}}\quad - \; \,\underbrace{\zeta _o \,\, p_o(S_o) \,\,\rho ,}_{\text {consumption by tumour cells}} \end{aligned}$$

14 $$\begin{aligned}&\frac{\partial S_g}{\partial t}\; = \;\underbrace{\beta _g\frac{\partial ^2 S_g}{\partial x^2}}_{\begin{array}{c} \text {diffusion} \end{array}} \quad - \;\underbrace{\lambda _g\,S_g}_{\begin{array}{c} \text {natural} \\ \text {decay} \end{array}}\quad - \;\underbrace{\zeta _g\,\, p_g(S_o,S_g) \,\,\rho }_{\text {consumption by tumour cells}} \end{aligned}$$

and

15 $$\begin{aligned} \frac{\partial S_l}{\partial t}\; = \;\underbrace{\beta _l\frac{\partial ^2 S_l}{\partial x^2}}_{\begin{array}{c} \text {diffusion} \end{array}} \quad - \; \underbrace{\lambda _l\,S_l}_{\begin{array}{c} \text {natural} \\ \text {decay} \end{array}}\quad + \;\,\underbrace{\zeta _l \,\, p_g(S_o,S_g)\,\,\rho ,}_{\text {production by tumour cells}} \end{aligned}$$

with $(t,x) \in (0,\mathrm{T}] \times (0,\mathrm{L})$, subject to suitable boundary conditions (see considerations below) and initial conditions.

In (13)–(15), the parameters $\beta _o>0$, $\beta _g>0$ and $\beta _l>0$ are the diffusion coefficients of oxygen, glucose and lactate, respectively, while the parameters $\lambda _o>0$, $\lambda _g>0$ and $\lambda _l>0$ are the natural decay rates of the three abiotic factors. The third term on the right-hand side of (13) is the consumption rate of oxygen by tumour cells, which is proportional to the product between the cell density $\rho$ and the rate of cell proliferation via oxidative phosphorylation $p_o(S_o)$, which is defined via (7). The parameter $\zeta _o>0$ is a conversion factor linked to oxygen consumption by the cells. Analogous considerations hold for the third term on the right-hand side of (14), which models the consumption rate of glucose by tumour cells. Furthermore, the third term on the right-hand side of (15) is the production rate of lactate by tumour cells, which is assumed to be proportional to the product between the cell density $\rho$ and the rate of cell proliferation via glycolysis $p_g(S_o,S_g)$ defined via (7). The constant of proportionality is the conversion factor $\zeta _l>0$ linked to lactate production by the cells.

We assume that oxygen and glucose enter the spatial domain through the blood vessel only, while lactate is flushed out through the blood vessel only. Hence, focussing on the case where the inflow rate of oxygen and glucose and the outflow rate of lactate are constant, we complement (13)–(15) with the following boundary conditions at $x=0$

16 $$\begin{aligned} S_o(t,0) = {\overline{S}}_o, \quad S_g(t,0) = {\overline{S}}_g, \quad S_l(t,0) = {\underline{S}}_l \quad \text {for all } t >0, \end{aligned}$$

where ${\overline{S}}_o>0$ and ${\overline{S}}_g>0$ are related to the average physiological levels of oxygen and glucose in proximity to blood vessels, while ${\underline{S}}_l>0$ is a small parameter of value close to zero. In particular, we have ${\overline{S}}_o>O_M$ and ${\underline{S}}_l < L_m$. Moreover, we assume that far from the blood vessel the concentrations of oxygen and glucose drop to some lower values $0<{\underline{S}}_o<{\overline{S}}_o$ and $0<{\underline{S}}_g<{\overline{S}}_g$, which correspond to the levels of oxygen and glucose, which are typically observed in regions distant from blood vessels. In particular, we have ${\underline{S}}_o<O_m$. Furthermore, we model abnormal accumulation of lactate, which is expected to occur far from blood vessels, imposing zero-flux boundary conditions. Therefore, we complement (13)–(15) with the following boundary conditions at $x=\mathrm{L}$

17 $$\begin{aligned} S_o(t,\mathrm{L}) = {\underline{S}}_o, \quad S_g(t,\mathrm{L}) = {\underline{S}}_g, \quad \dfrac{\partial S_l(t,\mathrm{L})}{\partial x} = 0 \quad \text {for all } t >0. \end{aligned}$$

## Main Results

In this section, we present the results of numerical simulations of the mathematical model defined by (4) coupled with (13)–(15) and we discuss their biological relevance. First, we describe the set-up of numerical simulations (see Sect. 3.1). Next, we present a sample of numerical solutions that summarise the spatial dynamics of tumour cells and abiotic factors (see Sect. 3.2). Then, we report on the results of numerical simulations showing the evolutionary dynamics of tumour cells and the emergence of phenotypic heterogeneity (see Sect. 3.3). Finally, we present the results of numerical simulations that reveal the existence of alternative evolutionary pathways that may lead to the development of resistance to hypoxia and acidity in vascularised tumours (see Sect. 3.4).

**Fig. 3:**
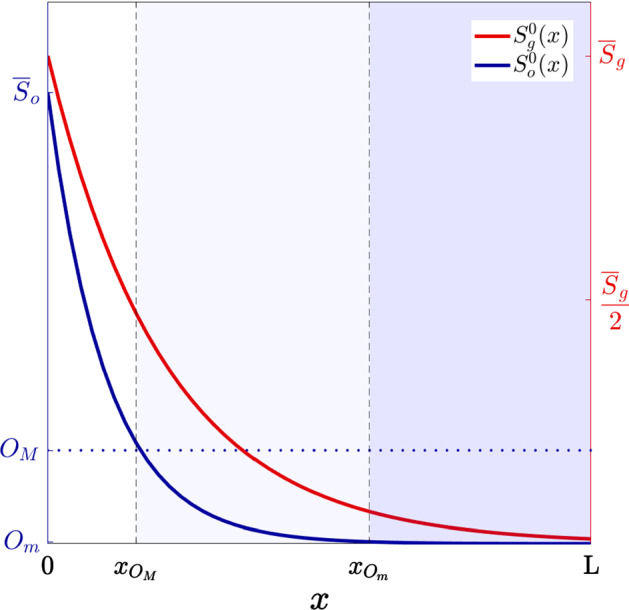
Plots of the initial distribution of oxygen $S^0_o(x)$ (blue curve, axis on the left) and the initial distribution of glucose $S^0_g(x)$ (red curve, axis on the right) which are defined in such a way as to match the experimental equilibrium distributions of oxygen and glucose presented in Molavian et al. (2009, Fig. 2). The vertical, dashed lines highlight the points $x_{O_M}$ and $x_{O_m}$ such that $S^0_o(x_{O_M})=O_M$ and $S^0_o(x_{O_m})=O_m$. Hence, the white region corresponds to normoxic conditions, the pale-blue region corresponds to moderately oxygenated environments and the blue region corresponds to hypoxic conditions (Color figure online)

### Set-Up of Numerical Simulations

Numerical simulations are carried out assuming $\mathrm{L}=400 \,\mu m$, which is chosen coherently with experimental data reported in Molavian et al. (2009), and $t \in [0,\mathrm{T}]$, where the final time $\mathrm{T}>0$ is such that the solutions of the model equations are at numerical equilibrium for $t=\mathrm{T}$.

Initial Conditions We consider (13), (14) and (15) subject, respectively, to the following initial conditions

18 $$\begin{aligned} S_o(0,x) = S^0_o(x), \quad S_g(0,x) = S^0_g(x) \quad \text {and} \quad S_l(0,x) = S^0_l(x) \equiv {\underline{S}}_l. \end{aligned}$$

Here, the functions $S^0_o(x)$ and $S^0_g(x)$ (see Fig. 3) are defined in such a way as to match the experimental equilibrium distributions of oxygen and glucose presented in Molavian et al. (2009, Fig. 2), while ${\underline{S}}_l$ is the same small parameter used in (16), i.e. ${\underline{S}}_l<L_m$. Initial conditions (18) correspond to a situation in which the initial distributions of oxygen and glucose match with experimental equilibrium distributions of such abiotic factors and lactate is present at a uniform level which is below the threshold level $L_m$, that is, the level below which the environment surrounding the cells is mildly acidic and the fittest level of expression of the acidity-resistant gene is the minimal one. Moreover, we complement (4) with the following initial condition

19 $$\begin{aligned} n(0,x,\mathbf{y}) = n^0(x,\mathbf{y}) := 200 \ \exp \bigg ({-\frac{(x-0)^2}{0.0002}-\frac{|\mathbf{y} - 0|^2}{0.4}}\bigg ), \end{aligned}$$

which corresponds to a biological scenario in which at the initial time $t=0$ most tumour cells are concentrated near the blood vessel and are characterised by the minimal expression level of both the hypoxia-resistant gene and the acidity-resistant gene.

Boundary Conditions We use the following values of the parameters ${\overline{S}}_o$, ${\overline{S}}_g$ and ${\underline{S}}_l$ in (16)

20 $$\begin{aligned} {\overline{S}}_o = 2.08 \times 10^{-6} \, \text {g/cm}^3, \quad {\overline{S}}_g=1.35 \times 10^{-4} \, \text {g/cm}^3, \quad {\underline{S}}_l= 10^{-8} \, \text {g/cm}^3 \end{aligned}$$

and the following values of the parameters ${\underline{S}}_o$ and ${\underline{S}}_g$ in (17)

21 $$\begin{aligned} {\underline{S}}_o = 2 \times 10^{-10}\, \text {g/cm}^3, \quad {\underline{S}}_g= 1.35 \times 10^{-6} \, \text {g/cm}^3. \end{aligned}$$

The values of ${\overline{S}}_o$ and ${\overline{S}}_g$ correspond to the average physiological levels of oxygen and glucose in proximity to blood vessels reported in Molavian et al. (2009). Moreover, the values of ${\underline{S}}_o$ and ${\underline{S}}_g$ correspond to the $0.1\%$ of ${\overline{S}}_o$ and the $1\%$ of ${\overline{S}}_g$, respectively. This is because, based on experimental data reported in Molavian et al. (2009), we expect the concentrations of oxygen and glucose at $400\,\upmu m$ from the blood vessel (i.e. at $x=\mathrm{L}$) to drop, respectively, below the $0.1\%$ and the $1\%$ of their value near the blood vessel.

Parameter Values Unless otherwise explicitly stated, we use the values of the model parameters listed in Table 1, which are chosen to be consistent with the existing literature, except for the values of the parameters $\eta _o$, $\eta _l$, $\lambda _l$ and $\zeta _l$ that are model specific in that we could not find them in the literature and are defined on the basis of the following considerations. The value of the conversion factor for lactate production $\zeta _l$ is chosen to be the same as the value of the conversion factor for glucose consumption $\zeta _g$. Furthermore, the value of the rate of natural decay of lactate $\lambda _l$ is such that the distribution of lactate at numerical equilibrium (i.e. the graph of $S_l(\mathrm{T},x)$) is similar to the lactate distributions reported in Molavian et al. (2009). Finally, although the values of the selection gradients $\eta _o$ and $\eta _l$ are chosen with exploratory aim, a systematic sensitivity analysis of the evolutionary dynamics of tumour cells to the values of these parameters was carried out, and the key findings from such sensitivity analysis are summarised by the results presented in Sect. 3.4. We also note that the value of the rate of phenotypic changes given in Table 1 is consistent with experimental data reported in Doerfler and Böhm (2006) and Duesberg et al. (2000).

**Table 1:** Parameter values used in numerical simulations

Par.	Biological meaning	Value	Units	References
$\beta _o$	Diffusion coefficient of oxygen	$1.46\,\times \,10^{-5}$	$\text {cm}^2 \,\text {s}^{-1}$	Molavian et al. (2009)
$\beta _g$	Diffusion coefficient of glucose	$1.10\,\times \,10^{-6}$	$\text {cm}^2 \,\text {s}^{-1}$	Molavian et al. (2009)
$\beta _l$	Diffusion coefficient of lactate	$1.9\,\times \,10^{-6}$	$\text {cm}^2\, \text {s}^{-1}$	Molavian et al. (2009)
$\beta _n$	Cell motility	$10^{-13}$	$\text {cm}^2 \,\text {s}^{-1}$	Villa et al. (2021)
$\theta$	Rate of phenotypic changes	$10^{-13}$	$\text {s}^{-1}$	Villa et al. (2021)
$\lambda _o$	Rate of natural decay of oxygen	$1.2\,\times \,10^{-3}$	$\text {s}^{-1}$	Molavian et al. (2009)
$\lambda _g$	Rate of natural decay of glucose	$2.33\,\times \,10^{-5}$	$\text {s}^{-1}$	Molavian et al. (2009)
$\lambda _l$	Rate of natural decay of lactate	$5\,\times \,10^{-2}$	$\text {s}^{-1}$	Ad hoc
$\gamma _o$	Max. prolif. rate via oxidative phosphorylation	$3.65\,\times \,10^{-7}$	$\text {s}^{-1}$	Molavian et al. (2009)
$\alpha _o$	Michaelis-Menten constant of oxygen	$6.4\,\times \,10^{-9}$	$\text {g cm}^{-3}$	Molavian et al. (2009)
$\gamma _g$	Max. prolif. rate via glycolysis	$3.42\,\times \,10^{-7}$	$\text {s}^{-1}$	Molavian et al. (2009)
$\alpha _g$	Michaelis–Menten constant of glucose	$9\,\times \,10^{-6}$	$\text {g cm}^{-3}$	Molavian et al. (2009)
$\eta _o$	Selection gradient related to oxygen	$3.65\,\times \,10^{-2}$	$\text {s}^{-1}$	Ad hoc
$\eta _l$	Selection gradient related to lactate	$10^{-2}$	$\text {s}^{-1}$	Ad hoc
$\kappa$	Rate of cell death due to competition for space	$2\,\times \,10^{-10}$	$\text {cm}^{3} \,\text {s}^{-1} \,\text {cells}^{-1}$	Lorenzi et al. (2018)
$\zeta _o$	Conversion factor for oxygen consumption	$10^{-8}$	$\text {g cells}^{-1}$	Lorenzi et al. (2018)
$\zeta _g$	Conversion factor for glucose consumption	$10^{-8}$	$\text {g cells}^{-1}$	Lorenzi et al. (2018)
$\zeta _l$	Conversion factor for lactate production	$10^{-8}$	$\text {g cells}^{-1}$	Ad hoc
$O_m$	Threshold level of oxygen for hypoxic env.	$8.2 \,\times \, 10^{-9}$	$\text {g cm}^{-3}$	Vaupel et al. (2001)
$O_M$	Threshold level of oxygen for normoxic env.	$4.3 \,\times \, 10^{-7}$	$\text {g cm}^{-3}$	Vaupel et al. (2001)
$L_m$	Threshold level of lactate for mildly acidic env.	$2 \,\times \, 10^{-5}$	$\text {g cm}^{-3}$	Robertson-Tessi et al. (2015)
$L_M$	Threshold level of lactate for highly acidic env.	$7.15 \,\times \, 10^{-5}$	$\text {g cm}^{-3}$	Robertson-Tessi et al. (2015)
$\mathrm{L}$	Max. distance from blood vessel	400	$\upmu$m	Molavian et al. (2009)

Numerical Methods Numerical solutions are constructed using a uniform discretisation of the interval $[0,\mathrm{L}]$ as the computational domain of the independent variable *x* and a uniform discretisation of the square $[0,1]^2$ as the computational domain of the independent variable $\mathbf{y}$. We also discretise the interval $[0,\mathrm{T}]$ with a uniform step. The method for constructing numerical solutions is based on an explicit finite difference scheme in which a three-point and a five-point stencils are used to approximate the diffusion terms in *x* and $\mathbf{y}$, respectively, and an implicit–explicit finite difference scheme is used for the reaction terms (LeVeque 2007; Lorz et al. 2013). All numerical computations are performed in Matlab.

### Dynamics of the Cell Density and the Concentrations of Abiotic Factors

**Fig. 4:**
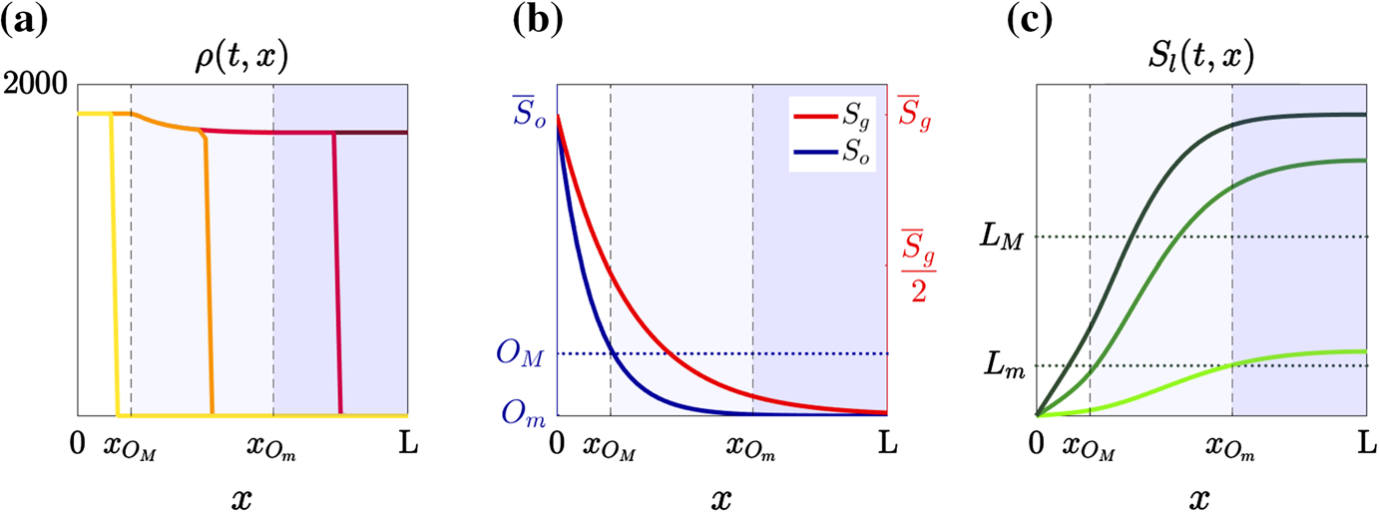
**a** Plots of the cell density $\rho (t,x)$ at four successive time instants (yellow, orange, red and burgundy lines). The burgundy line highlights $\rho (\mathrm{T},x)$. **b** Plots of the concentrations of oxygen $S_o(\mathrm{T},x)$ (blue line) and glucose $S_g(\mathrm{T},x)$ (red line). **c** Plots of the concentration of lactate $S_l(t,x)$ at three successive time instants (light-green, green and dark-green lines). The dark-green line highlights $S_l(\mathrm{T},x)$. In every panel, the vertical, dashed lines highlight the points $x_{O_M}$ and $x_{O_m}$ such that $S_o(\mathrm{T},x_{O_M})=O_M$ and $S_o(\mathrm{T},x_{O_m})=O_m$. Hence, the white region corresponds to normoxic conditions, the pale-blue region corresponds to moderately oxygenated environments and the blue region corresponds to hypoxic conditions (Color figure online)

The dynamics of the density of tumour cells and the concentrations of abiotic factors are illustrated by the plots in Fig. 4. In summary, the cell density and the concentration of lactate at successive times (i.e. the graphs of $\rho (t,x)$ for four different values of *t* and $S_l(t,x)$ for three different values of *t*) are displayed in Fig. 4a, c, respectively, while the concentrations of oxygen and glucose at time $\mathrm{T}$ (i.e. the graphs of $S_o(\mathrm{T},x)$ and $S_g(\mathrm{T},x)$) are displayed in Fig. 4b.

The dashed lines in Fig. 4 highlight the spatial positions $x_{O_M}$ and $x_{O_m}$ at which the oxygen concentration at time $\mathrm{T}$ crosses, respectively, the threshold values $O_M$ and $O_m$ (i.e. $S_o(\mathrm{T},x_{O_M})=O_M$ and $S_o(\mathrm{T},x_{O_m})=O_m$). Hence, the white region (i.e. the interval $[0,x_{O_M}]$), the pale-blue region (i.e. the interval $(x_{O_M},x_{O_m})$) and the blue region (i.e. the interval $[x_{O_m}, \mathrm{L}]$) correspond to normoxic, moderately oxygenated and hypoxic environmental conditions, respectively.

**Fig. 5:**
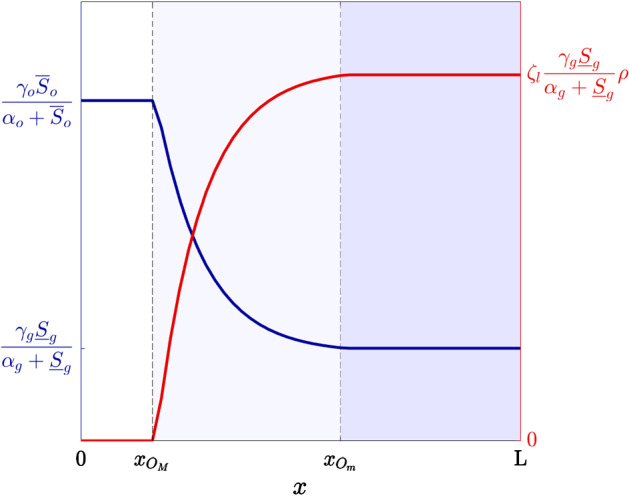
Plots of the cell proliferation rate $p_o(S_o(\mathrm{T},x))+p_g(S_o(\mathrm{T},x),S_g(\mathrm{T},x))$ (blue curve, axis on the left) and the production rate of lactate $\zeta _l \, p_g(S_o(\mathrm{T},x),S_g(\mathrm{T},x))\,\rho (\mathrm{T},x)$ (red curve, axis on the right) defined via (7). The vertical, dashed lines highlight the points $x_{O_M}$ and $x_{O_m}$ such that $S_o(\mathrm{T},x_{O_M})=O_M$ and $S_o(\mathrm{T},x_{O_m})=O_m$. Hence, the white region corresponds to normoxic conditions, the pale-blue region corresponds to moderately oxygenated environments and the blue region corresponds to hypoxic conditions (Color figure online)

The curves in Fig. 4a summarise the evolution of the cell number density $\rho (t,x)$, which behaves like an invading front whereby growth is saturated at a value that decreases with the distance from the blood vessel (i.e. $\rho (t,x)$ converges to a form of generalised transition wave Berestycki and Hamel 2012; Berestycki and Nadin 2020). These results illustrate how the synergistic interaction between cell proliferation, which occurs until the local carrying capacity of the tissue is reached, and cell movement allows tumour cells, which are initially located in the proximity of the blood vessel, to invade the surrounding tissue. The result that the plateau value of the cell number density decreases with the distance from the blood vessel, which is a result with broad structural stability under parameter changes (see Appendix A), reflects the fact that, in our model, the cell proliferation rate in normoxic conditions, whereby the energy needed for cell proliferation is produced via oxidative phosphorylation, is higher than the cell proliferation rate in moderately oxygenated environments, which in turn is higher than the cell proliferation rate in hypoxic conditions, whereby the energy needed for cell proliferation is produced via glycolysis (cf. the blue curve in Fig. 5). This is in line with experimental evidence, indicating that glycolysis is less efficient than oxidative phosphorylation as a mechanism to produce the energy required for cell proliferation.

Moreover, the curves in Fig. 4b show how the reaction–diffusion dynamics of oxygen and glucose, along with the inflow through the blood vessel and the consumption by tumour cells, lead the concentrations of such abiotic factors to converge to some stable values, which decrease as the distance from the blood vessel increases (i.e. at $t=\mathrm{T}$, $S_o(t,x)$ and $S_g(t,x)$ appear to be at numerical equilibrium and are monotonically decreasing functions of *x*). Notice that the distributions of oxygen and glucose at the final time $\mathrm{T}$ are close to the initial distributions $S^0_o(x)$ and $S^0_g(x)$ displayed in Fig. 3. This is to be expected. In fact, since $S^0_o(x)$ and $S^0_g(x)$ are defined in such a way as to match experimental equilibrium distributions of oxygen and glucose, under the biologically informed parameter values (cf. Table 1) and boundary conditions (cf. (16), (17) and (20), (21)) used here, the concentrations $S_o(t,x)$ and $S_g(t,x)$ reach quickly numerical equilibrium. We verified via additional numerical simulations (results not shown) that, as one would expect, the concentrations of oxygen and glucose at numerical equilibrium do not depend on the choice of the initial conditions.

Finally, the curves in Fig. 4c summarise the evolution of the concentration of lactate $S_l(t,x)$, which is the result of the interplay between the production of this abiotic factor by tumour cells, its reaction–diffusion dynamics and its outflow through the blood vessel. It is known that, as a waste product of glycolysis, lactate is mainly produced and accumulate in moderately oxygenated and hypoxic regions, where cell proliferation relies more on glycolysis and tissue perfusion is poorer. In agreement with this, the curves in Fig. 4c demonstrate that the concentration of lactate increases with the distance from the blood vessel. In particular, the values attained by $S_l(\mathrm{T},x)$, which depend on the values of the production rate of lactate in our model (cf. the red curve in Fig. 5), are in agreement with lactate concentrations reported in Molavian et al. (2009).

### Evolutionary Dynamics of Tumour Cells and Emergence of Phenotypic Heterogeneity

As discussed in the previous section, reaction–diffusion dynamics of abiotic factors and mutual interactions between abiotic factors and tumour cells lead to the emergence of spatial variations in the concentrations of oxygen and lactate—i.e. the oxygen concentration $S_o(\mathrm{T},x)$ is a monotonically decreasing function of *x*, while the lactate concentration $S_l(\mathrm{T},x)$ is a monotonically increasing function of *x* (cf. plots in Fig. 4b, c).

**Fig. 6:**
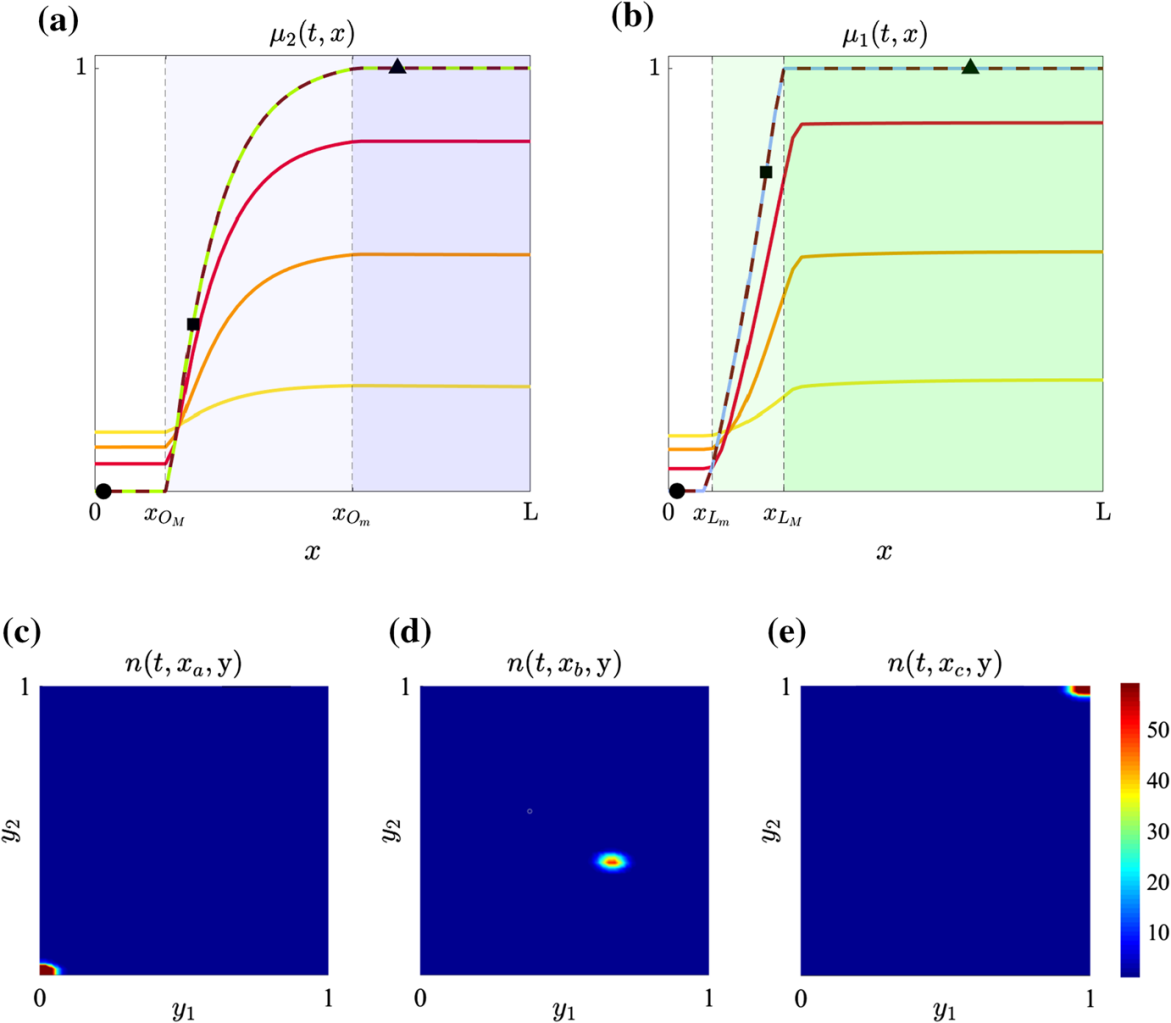
**a** Plots of the normalised level of expression of the hypoxia-resistant gene $\mu _2(t,x)$ at four successive time instants (yellow, orange, red and light-green lines). The light-green line highlights $\mu _2(\mathrm{T},x)$ and the burgundy, dashed line highlights the fittest level of expression of the hypoxia-resistant gene $\varphi _o(S_o(\mathrm{T},x))$ defined via (9). The vertical, dashed lines highlight the points $x_{O_M}$ and $x_{O_m}$ such that $S_o(\mathrm{T},x_{O_M})=O_M$ and $S_o(\mathrm{T},x_{O_m})=O_m$. Hence, the white region corresponds to normoxic conditions, the pale-blue region corresponds to moderately oxygenated environments and the blue region corresponds to hypoxic conditions. **b** Plots of the normalised level of expression of the acidity-resistant gene $\mu _1(t,x)$ at four successive time instants (yellow, orange, red and light-blue lines). The light-blue line highlights $\mu _1(\mathrm{T},x)$, and the burgundy, dashed line highlights the fittest level of expression of the acidity-resistant gene $\varphi _l(S_l(\mathrm{T},x))$ defined via (11). The vertical, dashed lines highlight the points $x_{L_m}$ and $x_{L_M}$ such that $S_l(\mathrm{T},x_{L_m})=L_m$ and $S_l(\mathrm{T},x_{L_M})=L_M$. Hence, the white region corresponds to mildly acidic conditions, the pale-green region corresponds to moderately acidic conditions and the green region corresponds to highly acidic conditions. **c**–**e** Plots of the local phenotypic distribution of tumour cells $n(\mathrm{T},x,\mathbf{y})$ at the points $x=x_a$, $x=x_b$ and $x=x_c$ highlighted, respectively, by the circle, square and triangle markers shown in **a** and **b** (Color figure online)

Spatial variability of oxygen and lactate concentrations can lead to the formation of environmental gradients resulting in the selection for cells with phenotypic characteristics that vary with distance from the blood vessel, thus promoting the emergence of intra-tumour phenotypic heterogeneity. The numerical results displayed in Fig. 6 support the idea that this can be effectively captured by our model.

The dashed lines in Fig. 6a, b highlight the fittest levels of expression of the hypoxia-resistant gene (see Fig. 6a) and the acidity-resistant gene (see Fig. 6b) at time $\mathrm{T}$ [i.e. the graphs of $\varphi _o(S_o(\mathrm{T},x))$ and $\varphi _l(S_l(\mathrm{T},x))$], while the solid lines display the local mean levels of expression of the hypoxia-resistant gene (see Fig. 6a) and the acidity-resistant gene (see Fig. 6b) at four successive times (i.e. the graphs of $\mu _2(t,x)$ and $\mu _1(t,x)$ for four different values of *t*). In analogy with Fig. 4, the vertical, dashed lines in Fig. 6a highlight the spatial positions $x_{O_M}$ and $x_{O_m}$ at which the oxygen concentration at time $\mathrm{T}$ crosses, respectively, the threshold values $O_M$ and $O_m$ (i.e. $S_o(\mathrm{T},x_{O_M})=O_M$ and $S_o(\mathrm{T},x_{O_m})=O_m$). Hence, the white region corresponds to normoxic conditions, the pale-blue region corresponds to moderately oxygenated environments and the blue region corresponds to hypoxic conditions. Moreover, the vertical, dashed lines in Fig. 6b highlight the spatial positions $x_{L_m}$ and $x_{L_M}$ at which the lactate concentration at time $\mathrm{T}$ crosses, respectively, the threshold values $L_m$ and $L_M$ (i.e. $S_l(\mathrm{T},x_{L_m})=L_m$ and $S_l(\mathrm{T},x_{L_M})=L_M$). Hence, the white region corresponds to mildly acidic conditions, the pale-green region corresponds to moderately acidic conditions and the green region corresponds to highly acidic conditions.

As shown by the plots in Fig. 6a, b, the local mean levels of expression of the hypoxia- and acidity-resistant genes converge, as time passes, to the fittest ones (i.e. $\mu _2(\mathrm{T},x)$ matches with $\varphi _o(S_o(\mathrm{T},x))$ and $\mu _1(\mathrm{T},x)$ matches with $\varphi _l(S_l(\mathrm{T},x)$), the values of which vary with the distance from the blood vessel depending on the local concentrations of oxygen and lactate. In more detail, the local mean level of expression of the hypoxia-resistant gene at time $\mathrm{T}$ is the minimal one in normoxic conditions (i.e. $\mu _2(\mathrm{T},x) \equiv 0$ for $x \in [0,x_{O_M}]$), the maximal one in hypoxic conditions (i.e. $\mu _2(\mathrm{T},x) \equiv 1$ for $x \in [x_{O_m},\mathrm{L}])$ and increases with the oxygen concentration in moderately oxygenated environments (i.e. $\mu _2(\mathrm{T},x)$ increases monotonically from 0 to 1 for $x \in (x_{O_M},x_{O_m})$). Furthermore, the local mean level of expression of the acidity-resistant gene at time $\mathrm{T}$ is the minimal one in the mildly acidic region (i.e. $\mu _1(\mathrm{T},x) \equiv 0$ for $x \in [0,x_{L_m}]$), the maximal one in highly acidic conditions (i.e. $\mu _1(\mathrm{T},x) \equiv 1$ for $x \in [x_{L_M},\mathrm{L}])$ and increases with the lactate concentration in moderately acidic environments (i.e. $\mu _1(\mathrm{T},x)$ increases monotonically from 0 to 1 for $x \in (x_{L_m},x_{L_M})$). These are results with broad structural stability under parameter changes (see Appendix A).

Finally, the plots in Fig. 6c–e show that, at every position $x \in [0,\mathrm{L}]$, the local phenotypic distribution of tumour cells at time $\mathrm{T}$ (i.e. the local population density function $n(\mathrm{T},x,\mathbf{y})$) is unimodal and attains its maximum at the fittest phenotypic state $\mathbf{y} = \big (\varphi _o(S_o(\mathrm{T},x)),\varphi _l(S_l(\mathrm{T},x) \big )$. As discussed in Appendix A, such a qualitative behaviour of $n(\mathrm{T},x,\mathbf{y})$ is in agreement with predictions based on the formal asymptotic results presented in Villa et al. (2021).

**Fig. 7:**
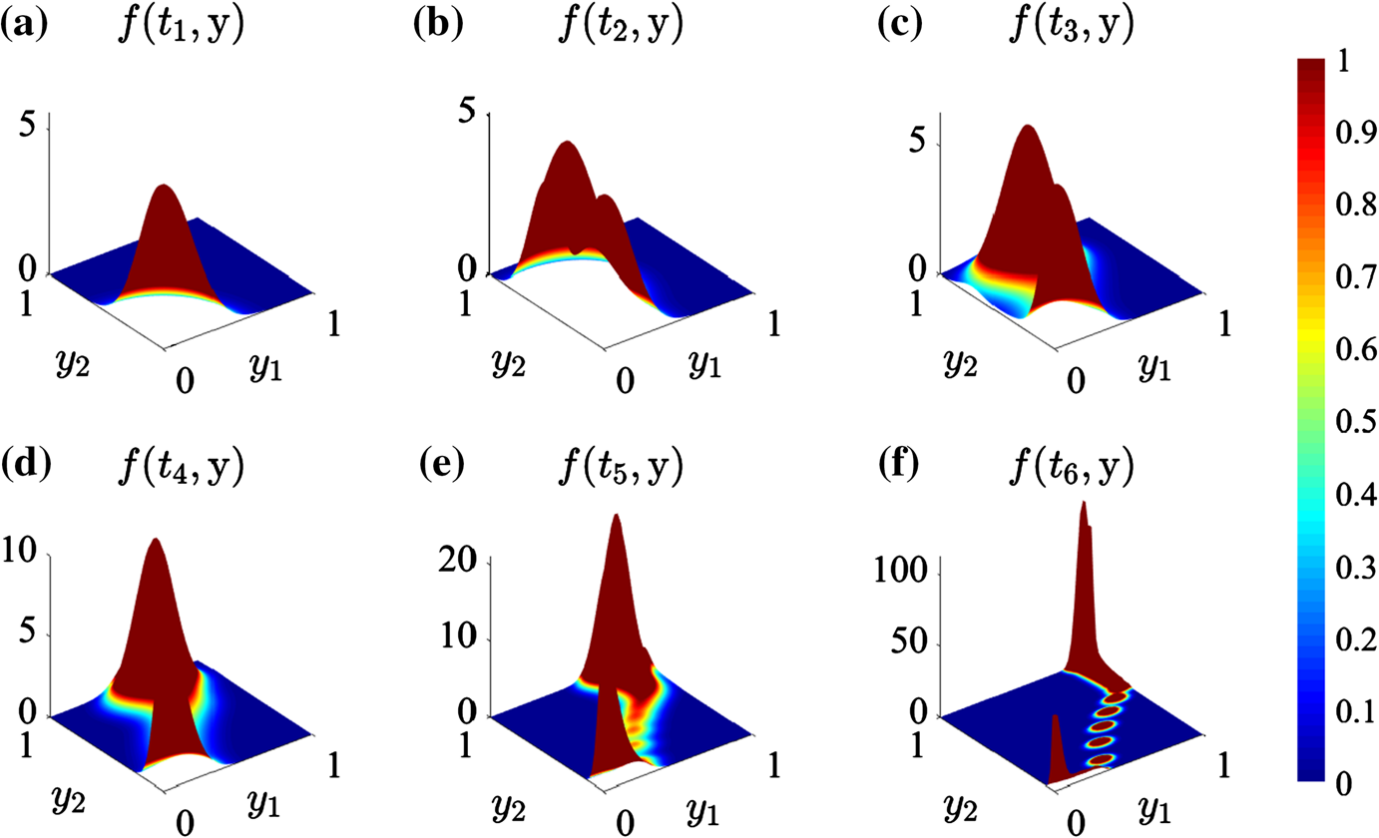
Plots of the phenotypic distribution of tumour cells across the whole tissue region $f(t,\mathbf{y})$ defined according to (2) at six successive time instants $t_1< t_2< \ldots < t_6$ (Color figure online)

The numerical results of Fig. 6 are complemented by the numerical results displayed in Fig. 7, which summarise the time evolution of the phenotypic distribution of tumour cells across the whole tissue region (i.e. the function $f(t,\mathbf{y})$ defined via (2)) and show that the maximum point of the distribution departs from the point $\mathbf{y}=(0,0)$ (i.e. the point corresponding to the minimal expression level of both the acidity-resistant gene and the hypoxia-resistant gene)—cf. the initial condition $n^0(x,\mathbf{y})$ defined via (19)—and moves toward the point $\mathbf{y}=(1,1)$, which corresponds to the maximal expression level of both the hypoxia-resistant gene and the acidity-resistant gene (i.e. the degree of malignancy of the tumour increases over time).

These results recapitulate key ideas about the eco-evolutionary dynamics of tumour cells proposed in Maley et al. (2017) by demonstrating how patchy resources (i.e. oxygen and glucose) and hazards (i.e. the selective pressure effects of lactate), which define the ecology of the tumour, can create multiple habitats whereby different phenotypic variants may be selected according to the principle of the “survival of the fittest” (i.e. through a functional trade-off between the ability to survive under certain environmental conditions and the evolutionary cost of acquiring such an ability). In the same vein, these results also support the idea that tumour growth can be conceptualised as an ecological process driven by consecutive phases of invasion and colonisation of new tissue habitats by tumour cells, which may be accelerated by the presence of gradients of abiotic factors corresponding to harsher environmental conditions.

### Alternative Evolutionary Pathways Leading to the Development of Resistance to Hypoxia and Acidity

In line with the classification of the evolutionary and ecological features of neoplasms presented in Maley et al. (2017), the ratio between the selection gradients $\eta _o$ and $\eta _l$ of our model could be seen as a measure of the impact that hypoxia and acidity may have on the eco-evolutionary dynamics of tumour cells. Hence, in this section we explore how the dynamics of the levels of expression of the acidity-resistant gene and the hypoxia-resistant gene across the whole tissue region (i.e. the functions $\nu _1(t)$ and $\nu _2(t)$ defined via (3)) are affected by the value of the ratio $\eta _o/\eta _l$.

**Fig. 8:**
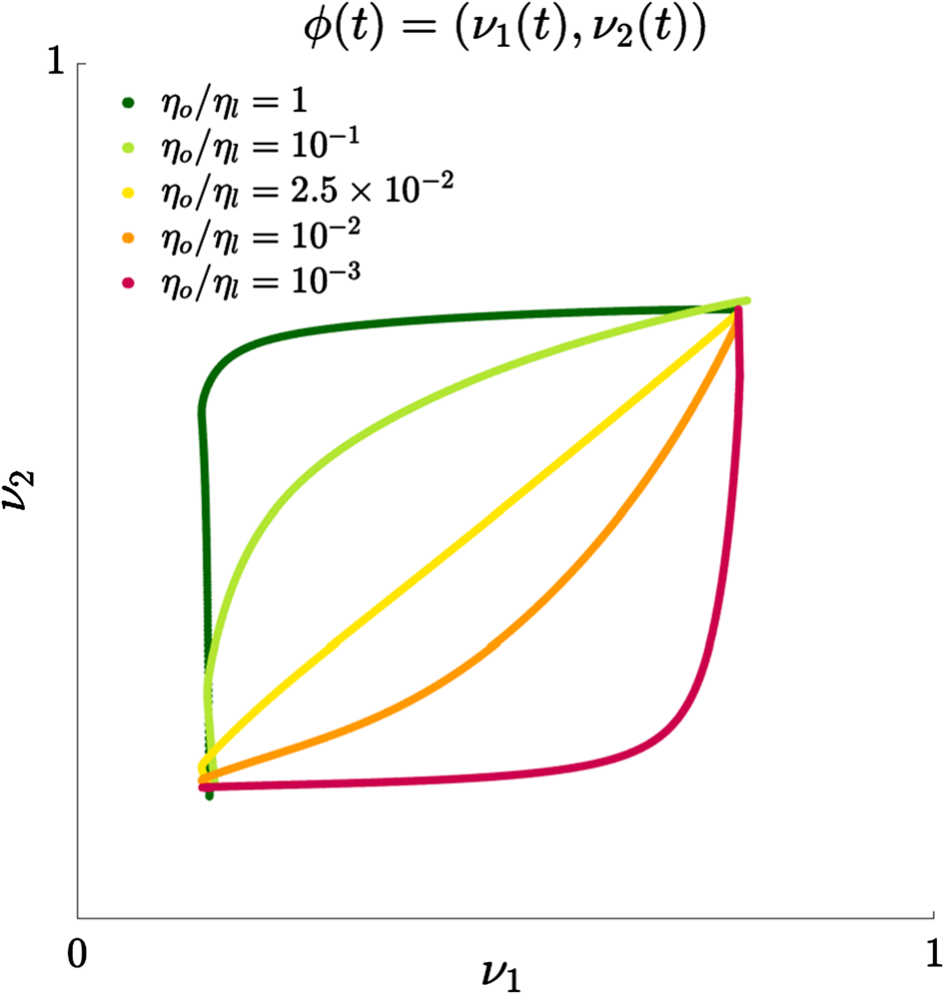
Plots of the curve $\phi (t) = \big (\nu _1(t), \nu _2(t) \big )$ for different values of the ratio between the selection gradient related to oxygen $\eta _o$ and the selection gradient related to lactate $\eta _l$. The functions $\nu _1(t)$ and $\nu _2(t)$ defined via (3) model the levels of expression of the acidity-resistant gene and the hypoxia-resistant gene across the whole tissue region, respectively (Color figure online)

The plot in Fig. 8, which displays the curve $\phi (t) = \big (\nu _1(t), \nu _2(t) \big )$, demonstrates that resistance to hypoxia and resistance to acidity arise via alternative evolutionary pathways depending on the value of $\eta _o/\eta _l$. Coherently with the results presented in the previous section, $\phi (t)$ departs from the point (0.15, 0.15) (i.e. the point corresponding to the initial levels of expression of the acidity-resistant gene and the hypoxia-resistant gene across the whole tissue region) and, for all values of $\eta _o/\eta _l$ considered, ultimately converges to a point corresponding to a high expression level of both the acidity-resistant gene and the hypoxia-resistant gene. However, larger values of the ratio $\eta _o/\eta _l$ lead the level of expression of the hypoxia-resistant gene $\nu _2(t)$ to increase faster than the level of expression of the acidity-resistant gene $\nu _1(t)$, while smaller values of $\eta _o/\eta _l$ correlate with a faster increase of $\nu _1(t)$ and a slower increase of $\nu _2(t)$. Furthermore, for intermediate values of the ratio $\eta _o/\eta _l$ we observe a simultaneous increase of the values of $\nu _1(t)$ and $\nu _2(t)$, whereas sufficiently large and sufficiently small values of $\eta _o/\eta _l$ correlate with a decoupling between the increase of $\nu _1(t)$ and $\nu _2(t)$. In more detail, if the ratio $\eta _o/\eta _l$ is sufficiently high, first $\nu _2(t)$ increases while $\nu _1(t)$ remains almost constant, and then, when $\nu _2(t)$ is sufficiently high, $\nu _1(t)$ starts increasing as well. On the other hand, in the case where $\eta _o/\eta _l$ is sufficiently low, we have that $\nu _1(t)$ increases first and then $\nu _2(t)$ starts increasing as soon as $\nu _1(t)$ becomes sufficiently high.

These results communicate the biological notion that: the strength of the selective pressures exerted by oxygen and lactate on tumour cells, which are quantified by the values of the selection gradients $\eta _o$ and $\eta _l$, may shape the emergence of hypoxic resistance and acidic resistance in tumours; the order in which such forms of resistance develop depends on the intensity of oxygen-driven selection in relation to the intensity of lactate-driven selection.

## Conclusions and Research Perspectives

In this work, we have developed a mathematical modelling approach to investigate the influence of hypoxia and acidity on the evolutionary dynamics of cancer cells in vascularised tumours.

The results of numerical simulations of a calibrated version of the model based on real data recapitulate the eco-evolutionary spatial dynamics of tumour cells and their adaptation to hypoxic and acidic microenvironments. In particular, the results obtained indicate that tumour cells characterised by lower levels of expression of hypoxia- and acidity-resistant genes are to be expected to colonise well-oxygenated and mildly acidic regions of vascularised tissues, whereas cells expressing a more aggressive phenotype characterised by higher levels of resistance to hypoxia and acidity will ultimately populate tissue regions corresponding to hypoxic and acidic microenvironments. Such theoretical findings recapitulate histological data on ductal carcinoma *in situ*, showing that the levels at which the acidity-resistant gene LAMP2 and the hypoxia-resistant gene GLUT-1 are expressed by cancer cells increase moving from the walls to the centre of the milk duct (i.e. moving from more oxygenated and less acidic regions to regions that are less oxygenated and more acidic) (Damaghi et al. 2015; Gatenby et al. 2007).

Moreover, our theoretical findings reconcile the conclusions of Gatenby et al. (2007), suggesting that tumour cells acquire first resistance to hypoxia and then resistance to acidity, and the conclusions of Robertson-Tessi et al. (2015), supporting the idea that the two forms of resistance are acquired in reverse order, by showing that the order in which resistance to hypoxia and resistance to acidity arise depend on the ways in which oxygen and lactate act as environmental stressors in the evolutionary dynamics of tumour cells, which are known to vary between tissue types and between patients (Maley et al. 2017).

We conclude with a brief overview of possible research perspectives. Along the lines of Lorenzi et al. (2021), the modelling framework presented here could be extended to incorporate additional details of cell movement and mechanical interactions between cells (Ambrosi and Preziosi 2002; Astanin and Preziosi 2008; Byrne and Preziosi 2003), which would make it possible to investigate the interplay between phenotypic evolution of cancer cells and tumour growth. Alternative ways of modelling the effect of heritable, spontaneous phenotypic changes, for instance via integral terms (Barles et al. 2009; Busse et al. 2020; Calsina et al. 2013; Diekmann et al. 2005; Lorz et al. 2013), could also be considered, in order to capture finer details of the processes that drive phenotypic changes (Amadori et al. 2015, 2018; Carja and Plotkin 2017; Champagnat et al. 2006; Di Costanzo et al. 2018). Moreover, it would be relevant to include in the model the effects of stress-induced phenotypic changes caused by hypoxia and acidity, which might be taken into account by introducing a drift term in the balance equation for the local population density function of tumour cells, as similarly done in Celora et al. (2021), Chisholm et al. (2015), Lorenzi et al. (2015). In the vein of Alfaro and Veruete (2019), Lorenzi and Pouchol (2020), Michod et al. (2006), Nguyen et al. (2019), it would also be interesting to consider possible generalisations of the definition of the fitness function employed here, for instance by letting the selection gradients depend on the concentrations of the abiotic factors, which would allow the concavity of the fitness function to vary across the tumour depending on the local environmental conditions, and by introducing multiple fitness peaks, in order to explore alternative ways in which the spatial and evolutionary dynamics of tumour cells, and their dynamical interactions with abiotic factors, may drive the emergence of intra-tumour phenotypic heterogeneity (Gerlinger et al. 2012).

Building on Ardaševa et al. (2020a), it would be interesting to generalise the model to study the effects of fluctuations in the inflow rate of oxygen and glucose and the outflow rate of lactate in the evolution of cancer cells. In fact, when in hypoxic conditions, cancer cells are known to produce and secrete proangiogenic factors which induce the formation of new blood vessels departing from existing ones. Such an angiogenic process results in the formation of a disordered tumour vasculature whereby the rates at which oxygen and glucose enter the tumour and the rate at which lactate is flushed out through intra-tumour blood vessels fluctuate over time, which impacts on the evolutionary dynamics of cancer cells (Dewhirst 2009; Kimura et al. 1996; Matsumoto et al. 2010; Michiels et al. 2016).

It would also be interesting to extend the model in order to investigate the role of phenotypic transitions triggered by hypoxia and acidity—such as the epithelial–mesenchymal transition induced by hypoxic environmental conditions (Misra and Pandey 2012; Tam et al. 2020; Zhang et al. 2015) and the acquisition of the metastatic phenotype promoted by acidic microenvironments (Damaghi et al. 2015; DeClerck and Elble 2010; Fais et al. 2014)—in the phenotypic adaptation of cancer cells and tumour growth.

Furthermore, since resistance to hypoxia is known to correlate with resistance to chemotherapy and radiotherapy (Cosse and Michiels 2008; DeClerck and Elble 2010; Lewin et al. 2018; Prokopiou et al. 2015; Teicher 1994), building on Chaplain et al. (2021); Lorenzi et al. (2018); Lorz et al. (2015), it would be relevant for anti-cancer therapy to address numerical optimal control for an extended version of the model that takes into account the effect of chemotherapy and/or radiotherapy (Almeida et al. 2019; Pouchol et al. 2018), which could inform the development of optimised cancer treatment protocols that exploit evolutionary and ecological principles (Acar et al. 2020; Gatenby et al. 2009; Korolev et al. 2014; Merlo et al. 2006).

Finally, it would certainly be interesting to extend the model presented here to two- and three-dimensional spatial domains. This would make it possible to explore the evolutionary dynamics of tumour cells in a broader range of biological and clinical scenarios and, in particular, it would allow to investigate how the level of tissue vascularisation and the distribution of blood vessels may affect the eco-evolutionary process leading to the emergence of resistance to hypoxia and acidity in vascularised tumours.

## Figures and Tables

**Fig. 9:**
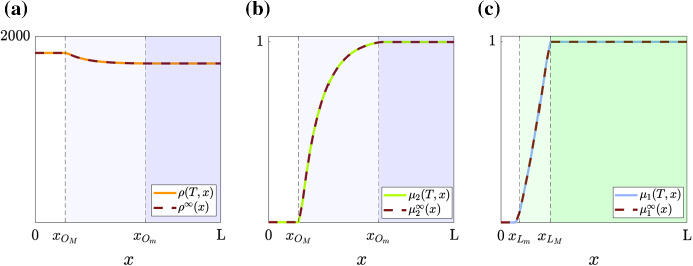
**a** Comparison between $\rho ^{\infty }(x)$ defined via (24) (dashed line) and $\rho (\mathrm{T},x)$ displayed in Fig. 4 (solid line). **b** Comparison between $\mu ^{\infty }_2(x)$ defined via (25) (dashed line) and $\mu _2(\mathrm{T},x)$ displayed in Fig. 6 (solid line). **c** Comparison between $\mu ^{\infty }_1(x)$ defined via (25) (dashed line) and $\mu _1(\mathrm{T},x)$ displayed in Fig. 6 (solid line). Here, $\rho ^{\infty }(x)$, $\mu ^{\infty }_1(x)$ and $\mu ^{\infty }_2(x)$ are computed defining $S_o^{\infty }(x)$, $S_g^{\infty }(x)$ and $S_l^{\infty }(x)$ as $S_o(\mathrm{T}, x)$, $S_g(\mathrm{T}, x)$ and $S_l(\mathrm{T}, x)$ displayed in Fig. 4, and using the same parameter values as those used to obtain the numerical results of Figs. 4 and 6 (cf. Table 1) (Color figure online)

## Steady-State Properties of the Model for the Dynamics of Tumour Cells and Robustness of the Results Obtained

Steady-State Properties of the model for the dynamics of tumour cells Using a time scale separation approach and a formal asymptotic method similar to those employed in Villa et al. (2021), one can formally show that when spontaneous phenotypic changes and undirected, random cell movement occur on slower time scales compared to cell division and death, as in the case of this work (*cf.* the parameter values listed in Table 1), and the fitness function $R(S_o,S_g,S_l,\rho , y_1, y_2)$ is a strictly concave function of $\mathbf{y}=(y_1,y_2)$ of the form considered here, i.e.

22 $$\begin{aligned} R\,(S_o,S_g,S_l,\rho , y_1, y_2) := p\,(S_o,S_g,S_l,y_1, y_2) - \kappa \rho \end{aligned}$$

with

23 $$\begin{aligned} p\,(S_o,S_g,S_l,y_1, y_2):= & {} \dfrac{\gamma _o\,S_o}{\alpha _o+S_o}\,\big (1-\varphi _o(S_o)\big ) + \dfrac{\gamma _g\,S_g}{\alpha _g+S_g}\,\varphi _o(S_o) \nonumber \\&-\eta _o \, \big (y_2-\varphi _o(S_o)\big )^2-\eta _l \, \big (y_1-\varphi _l(S_l)\big )^2 \end{aligned}$$

where $\varphi _o: {\mathbb {R}}^+ \rightarrow [0,1]$ and $\varphi _l: {\mathbb {R}}^+ \rightarrow [0,1]$, the steady-state distribution of tumour cells, $n^{\infty }(x,y_1,y_2)$, will be unimodal and such that the steady-state cell density,

$$\begin{aligned} \rho ^{\infty }(x) = \int _{0}^1 \int _{0}^1 n^{\infty }(x, y_1, y_2) \, \mathrm{d}y_1 \, \mathrm{d}y_2, \end{aligned}$$

the steady-state local mean level of expression of the acidity-resistant gene,

$$\begin{aligned} \mu ^{\infty }_1(x) =\frac{1}{\rho ^{\infty }(x)} \int _{0}^1 \int _{0}^1 y_1 \,n^{\infty }(x, y_1, y_2) \, \mathrm{d}y_1 \, \mathrm{d}y_2, \end{aligned}$$

and the steady-state local mean level of expression of the hypoxia-resistant gene,

$$\begin{aligned} \mu ^{\infty }_2(x) =\frac{1}{\rho ^{\infty }(x)} \int _{0}^1 \int _{0}^1 y_2 \,n^{\infty }(x, y_1, y_2)\, \mathrm{d}y_1 \, \mathrm{d}y_2, \end{aligned}$$

will satisfy the following system

$$\begin{aligned} {\left\{ \begin{array}{ll} R\Big (S_o^{\infty }(x),S_g^{\infty }(x),S_l^{\infty }(x),\rho ^{\infty }(x),\mu ^{\infty }_1(x), \mu ^{\infty }_2(x)\Big )=0, \\ \\ \dfrac{\partial R}{\partial y_1}\Big (S_o^{\infty }(x),S_g^{\infty }(x),S_l^{\infty }(x),\rho ^{\infty }(x),\mu ^{\infty }_1(x), \mu ^{\infty }_2(x)\Big )=0, \\ \\ \dfrac{\partial R}{\partial y_2}\Big (S_o^{\infty }(x),S_g^{\infty }(x),S_l^{\infty }(x),\rho ^{\infty }(x),\mu ^{\infty }_1(x), \mu ^{\infty }_2(x)\Big )=0, \end{array}\right. } \quad \forall x \in [0,\mathrm{L}], \end{aligned}$$

where $S_o^{\infty }(x)$, $S_g^{\infty }(x)$ and $S_l^{\infty }(x)$ are the steady-state concentrations of oxygen, glucose and lactate, respectively.

Substituting definitions (22) and (23) into the above system and solving for $\rho ^{\infty }$, $\mu ^{\infty }_1$ and $\mu ^{\infty }_2$ yields

24 $$\begin{aligned} \rho ^{\infty }(x)= \dfrac{1}{\kappa } \left( \dfrac{\gamma _o\,S_o^{\infty }(x)}{\alpha _o+S_o^{\infty }(x)}\,\big (1-\varphi _o(S_o^{\infty }(x))\big ) + \dfrac{\gamma _g\,S_g^{\infty }(x)}{\alpha _g+S_g^{\infty }(x)} \, \varphi _o(S_o^{\infty }(x))\right) \end{aligned}$$

and

25 $$\begin{aligned} \mu ^{\infty }_1(x) = \varphi _l(S_l^{\infty }(x)), \quad \mu ^{\infty }_2(x) = \varphi _o(S_o^{\infty }(x)). \end{aligned}$$

These formal results are confirmed both by the plots in Fig. 6c–e, which show that, at every position $x \in [0,\mathrm{L}]$, the local phenotypic distribution of tumour cells at the end of numerical simulations $n(\mathrm{T},x,\mathbf{y})$ is unimodal, and by the plots in Fig. 9, which demonstrate that, defining $S_o^{\infty }(x)$, $S_g^{\infty }(x)$ and $S_l^{\infty }(x)$ as $S_o(\mathrm{T}, x)$, $S_g(\mathrm{T}, x)$ and $S_l(\mathrm{T}, x)$ displayed in Fig. 4, respectively, and using the same parameter values as those used to obtain the numerical results of Figs. 4 and 6, there is an excellent quantitative match between: $\rho ^{\infty }(x)$ defined via (24) and $\rho (\mathrm{T},x)$ displayed in Fig. 4; $\mu ^{\infty }_1(x)$ defined via (25) and $\mu _1(\mathrm{T},x)$ displayed in Fig. 6; $\mu ^{\infty }_2(x)$ defined via (25) and $\mu _2(\mathrm{T},x)$ displayed in Fig. 6.

Robustness of the Results Obtained From the form of the steady-state cell density $\rho ^{\infty }$ given by (24), the form of the steady-state local mean level of expression of the acidity-resistant gene $\mu ^{\infty }_1$ given by (25), and the form of the steady-state local mean level of expression of the hypoxia-resistant gene $\mu ^{\infty }_2$ given by (25), one can see that the qualitative properties of the results presented in Sects. 3.2 and 3.3—i.e. the fact that:

(i) the plateau value of the cell density $\rho$ decreases with the distance from the blood vessel;
(ii) the local mean level of expression of the acidity-resistant gene $\mu _1$ at equilibrium is the minimal one in mildly acidic regions, the maximal one in highly acidic conditions and increases with the lactate concentration in moderately acidic environments;
(iii) the local mean level of expression of the hypoxia-resistant gene $\mu _2$ at equilibrium is the minimal one in normoxic conditions, the maximal one in hypoxic conditions and increases with the oxygen concentration in moderately oxygenated environments
  - remain intact under a broad range of parameter values.
